# PCLAF promotes neuroblastoma G1/S cell cycle progression via the E2F1/PTTG1 axis

**DOI:** 10.1038/s41419-022-04635-w

**Published:** 2022-02-24

**Authors:** Xiaowei Liu, Yuanxia Cai, Cheng Cheng, Yaoyao Gu, Xiaoxiao Hu, Kai Chen, Yeming Wu, Zhixiang Wu

**Affiliations:** 1 Department of Pediatric Surgery, Xinhua Hospital, School of Medicine, Shanghai Jiaotong University, Shanghai, 200092 China; 2 Division of Pediatric Oncology, Shanghai Institute of Pediatric Research, Shanghai, 200092 China; 3 Department of Pediatric Surgery, Children’s Hospital of Soochow University, Suzhou, 215003 China

**Keywords:** Cancer epigenetics, Cell growth

## Abstract

PCLAF (PCNA clamp-associated factor), also known as PAF15/ KIAA0101, is overexpressed in most human cancers and is a predominant regulator of tumor progression. However, its biological function in neuroblastoma remains unclear. PCLAF is extremely overexpressed in neuroblastoma and is associated with poor prognosis. Through the analysis of various data sets, we found that the high expression of *PCLAF* is positively correlated with increased stage and high risk of neuroblastoma. Most importantly, knocking down PCLAF could restrict the proliferation of neuroblastoma cells in vitro and in vitro. By analyzing RNA-seq data, we found that the enrichment of cell cycle-related pathway genes was most significant among the differentially expressed downregulated genes after reducing the expression of *PCLAF*. In addition, PCLAF accelerated the G1/S transition of the neuroblastoma cell cycle by activating the E2F1/PTTG1 signaling pathway. In this study, we reveal the mechanism by which PCLAF facilitates cell cycle progression and recommend that the PCLAF/E2F1/PTTG1 axis is a therapeutic target in neuroblastoma.

## Introduction

Neuroblastoma, a malignant tumor originating in the sympathetic nervous system, is the most common extracranial tumor in childhood [1–3]. The primary clinical manifestation of neuroblastoma is the appearance of tumor masses of different sizes in the adrenal glands or sympathetic ganglia, and the clinical prognosis largely hinges on the stage of the disease [4, 5]. Treatment strategies for neuroblastoma are tailored according to the risk classification of the disease as well as clinical and tumor biological markers [6]. Patients in the low-risk group often relapse spontaneously and are ordinarily treated with observation and surgical resection. For high-risk cancer patients, exhaustive medical treatment is required. Therefore, surviving patients frequently are subjected to multitudinous sequelae, and the recurrence rate of high-risk cases reaches up to 50%, which cannot be cured once they relapse [7, 8]. Therefore, we need to explore the aberrant signaling pathways in neuroblastoma to screen appropriate inhibitors to replace conventional chemotherapy, which will diminish the dose required for drug treatment and reduce the toxic side effects. Comprehending the underlying molecular biological mechanisms of neuroblastoma is indispensable for exploring up-and-coming strategies to enhance prognostic stratification and further ameliorate the prognosis of patients.

PCLAF (PCNA clamp-associated factor), also known as PAF15/ KIAA0101, was determined as a PCNA-binding partner. PCLAF is a nucleoprotein with a molecular weight of 15 kDa that was initially identified by yeast two-hybrid screening and can bind to PCNA (proliferating cell nuclear antigen) [9]. As one of the proteins that can interact with PCNA, the role of PCLAF is closely associated with the function of PCNA. PCNA acts as a cofactor of the DNA polymerase delta during binding to the DNA template. PCLAF is also one such cofactor and can regulate the binding of DNA polymerase and PCNA [10, 11]. Many reports have shown that PCLAF also has a multitude of essential biological functions. For example, it participates in DNA damage repair to affect cell survival, is involved in cell cycle regulation, and affects cell proliferation. PCLAF silencing can reduce the proliferative ability of anaplastic thyroid cancer and cervical cancer cells, prevent the DNA synthesis and colony formation of pancreatic cancer and adrenal cancer cells, and augment the number of G0/G1 cells in adrenal cancer and cervical cancer cells [12–17]. In this study, we demonstrated that PCLAF is highly expressed in neuroblastoma, which can accelerate neuroblastoma cell proliferation and cell cycle progression and restrain cell apoptosis, and is related to the poor prognosis of patients. In the cell cycle signaling pathway, the mRNA level of *PTTG1* decreases most significantly after PCLAF expression is downregulated. At the same time, our analysis of neuroblastoma databases and clinical tissue samples confirmed that PCLAF showed a significantly positive correlation with PTTG1. Subsequent mechanistic studies confirmed that PCLAF could stimulate the expression of PTTG1, affecting the G1-S transition of neuroblastoma cells. Subsequently, we found that E2F1 mediates PCLAF-induced PTTG1 expression, and our database analysis showed a positive correlation between E2F1, PCLAF, and PTTG1. In conclusion, we clarify the existence of the PCLAF/E2F1/PTTG1 axis, which has an imperative role in cell cycle progression in neuroblastoma cells.

## Materials and Methods

### Tissue Specimens

In our study, a total of 75 patients with primary neuroblastomas that had a pathological diagnosis were selected from September 2012 to February 2015 at the Xinhua Hospital affiliated to Shanghai Jiaotong University School of Medicine. See the Supplementary Material for details.

### Tissue microarray and immunohistochemistry (IHC)

The selected tissue samples were divided into tiny parts, fixed in 4% paraformaldehyde overnight, trimmed, and embedded in paraffin according to the arrangement of the experimental plan. As previously described [18, 19], see the Supplementary Material for details.

### Cell culture and transfection

Human neuroblastoma cell lines SK-N-BE (2), IMR-32, SH-SY5Y, and SK-N-SH were purchased from the Chinese Academy of Sciences. SK-N-AS was obtained from the American Type Culture Collection (ATCC). See the Supplementary Material for details.

### Lentivirus-mediated silencing of *PCLAF* and overexpression of *E2F1*

Lentivirus, including complementary oligonucleotide sequences (Table S1) and non-target control shRNA (NC), were established and synthesized by Genomeditech (Shanghai, China). At 72 h post-transfection, 2 μg/ml puromycin (Cat. #ST551; Beyotime, China) was added to select for the stably transfected cell lines. A E2F1 overexpressing lentivirus with Flag was purchased from Genomeditech (Shanghai, China). E2F1 overexpression cell lines were constructed in SK-N-BE (2) and SH-SY5Y cells.

### Animal experiments

Six-week-old female nude mice were obtained from Shanghai Slack Laboratory Animal Co., Ltd., China. All experiments were approved by the Animal Care and Use Committee of Xinhua Hospital, and all procedures have been described previously [18], see the Supplementary Material for details.

### Cell viability analysis

Cell Counting Kit-8 (CCK-8) (Yeasen, Shanghai, China) was used to analyze cell viability. See the Supplementary Material for details.

### EDU incorporation test

For flow cytometry analysis of the proliferating cells, a Cell-Light EdU Apollo 488 In Vitro Flow Cytometry Kit (RiboBio, Guangzhou, China) was used to examine EdU-positive cells according to the manufacturer’s protocol. The fluorescence signal at 488 nm was measured with a flow cytometer.

### Cell cycle detection

Using the Cell Cycle and Apoptosis Analysis Kit (C1052), the cells were collected and stained with propidium iodide according to the manufacturer’s instructions. Then the cell cycle was measured by flow cytometry.

### Annexin V-FITC/propidium iodide (PI) flow cytometry

SK-N-BE (2) and SH-SY5Y cells were plated in a 6-well plate and transfected with si*PCLAF* after 24 h. After 48 h, an Annexin V-FITC kit (BD Biosciences, San Diego, CA, USA) was used to examine cell apoptosis according to the manufacturer’s protocol.

### Western blotting

Western blotting was implemented as mentioned previously [18]. See the Supplementary Material for details.

### Quantitative real-time PCR

See the Supplementary Material for details.

### *PCLAF*-related gene enrichment analysis

The “Similar Gene Detection” module of GEPIA2 was analyzed to acquire the top 100 *PCLAF*-related target genes based on all TCGA tumor and typical tissue data sets.

See the Supplementary Material for details.

### RNA sequencing analysis

SK-N-BE (2) cells were stably transfected with Sh*PCLAF* and shNC, and 2 µg total RNA of each sample was extracted using TRIzol reagent (Thermo Fisher Scientific, USA). See the Supplementary Material for details.

### Chip sequencing analysis process

Chip sequencing data were obtained from Gene Expression Omnibus (GEO) or Encyclopedia of DNA Elements (ENCODE) (Table S3). See the Supplementary Material for details.

### Neuroblastoma data set analysis

The neuroblastoma patient data sets in the R2 Genomics Analysis and Visualization Platform (http://r2.amc.nl) were used for analyses of clinical information. Four publicly available data sets were analyzed: Kocak (GEO: GSE45547), SEQC (GEO: GSE49710), NRC (GEO: GSE85047), and Fischer (GEO: GSE120572).

### Statistical analysis

All analyses were performed using GraphPad Prism 8 (GraphPad Software, Inc, La Jolla, CA, USA) and Windows version SPSS 25.0 software (SPSS, Chicago, IL, USA). Pearson’s chi-squared test and Fisher’s exact test were performed for qualitative data and Student’s t-test (two-tailed) for quantitative data. Correlations were examined using the Spearman rank correlation. The survival rate was analyzed by Kaplan–Meier analysis. Two-tailed Student’s t-test was used for group comparisons. The effects of age at diagnosis, clinical stage, high-risk, and *MYCN* amplification as prognostic factors for clinical outcome were measured by stepwise multivariate Cox regression analysis. The results were expressed as the mean ± standard error of the mean (SEM). A *p* value of < 0.05 was considered to indicate a statistically significant difference. Significance was expressed as: *p < 0.05, **p < 0.01, ***p < 0.001, and ****p < 0.0001.

## Results

### High expression of PCLAF in neuroblastoma is a significant adverse prognostic factor

**Fig. 1:**
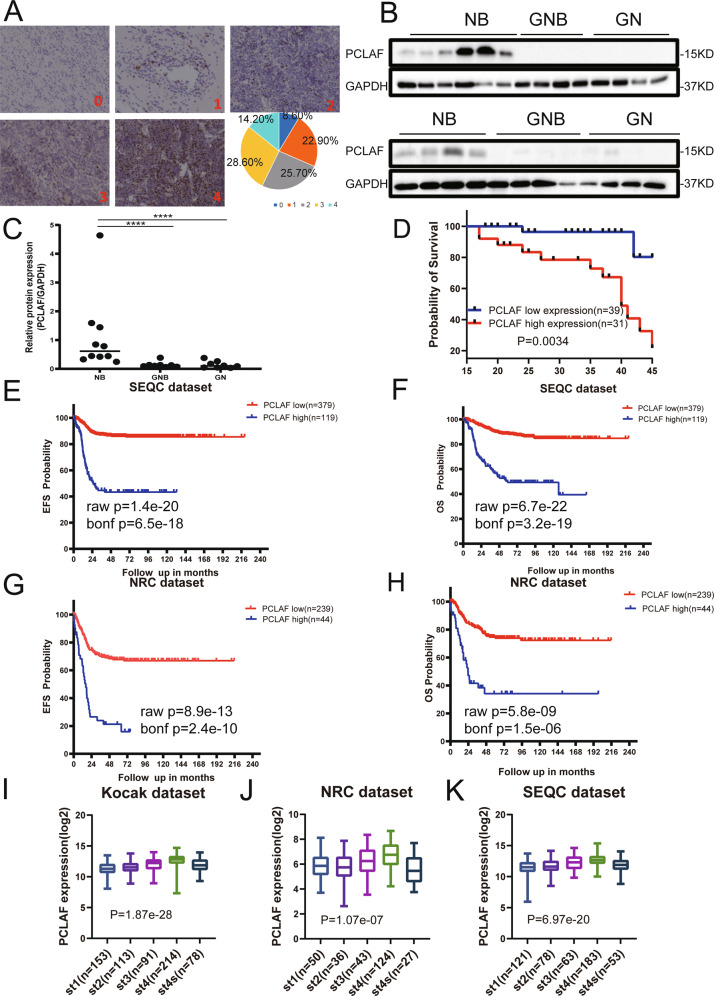
Expression and roles of PCLAF in neuroblastoma. (**A**) Representative pictures of distinct levels (0–4) of IHC staining of PCLAF and the proportions of five levels; (**B, C**) 10NB, 8GNB and 8GN tumor tissue proteins extracted for Western Blot to discover PCLAF expression, and Image J was used to quantify protein bands. The data in (**C**) were analyzed by Mann-Whitney *U*-test (*n* = 28); (**D**) Kaplan-Meier analysis of OS in TMA of 70 neuroblastoma samples based on PCLAF expression with the log-rank test *P* value indicated; (**E**–**H**) Kaplan-Meier analysis of OS and EFS for the SEQC data set (*n* = 498) and the NRC data set (*n* = 275), based on PCLAF expression with the log-rank test *p* value indicated; (**I**–**K**) PCLAF expression levels in stage (St) 1-4 S tumors was show in box plot. (*p < 0.05, ***p < 0.001and ****p < 0.0001, and bar graphs represent the mean ± SEM).

To determine whether PCLAF has potential clinical significance in neuroblastoma, PCLAF IHC was performed on a neuroblastoma TMA. We enrolled 70 cases for the subsequent analysis. Among the 70 neuroblastoma samples, 41 were from male patients, and 29 were from female patients. The IHC results showed that PCLAF was predominantly distributed in the nuclei, and the expression of PCLAF differed in neuroblastoma of distinct pathological types. A semi-quantitative grading system (0 to 4) was implemented on the basis of the number of positive cells: grade 0 (8.60%), grade 1 (22.90%), grade 2 (25.70%), grade 3 (28.60%), and grade 4 (14.20%) (Fig. 1A, Supplementary Fig. 1A). Next, we selected frozen fresh samples for western blot analysis to confirm further that the expression of PCLAF was significantly higher in neuroblastoma patients compared with and GNB (ganglioneuroblastoma) and GN (ganglioneuroma)patients (Fig. 1B, C). On the basis of the TMA data, we evaluated the relationship between PCLAF expression and 3-year overall survival (OS). Kaplan–Meier analysis demonstrated that patients in the high PCLAF expression group were significantly correlated with unfavorable OS than those with low expression of PCLAF (*p* = 0.0034; Fig. 1D). Considering the size of the sample, to further explore the clinical significance of *PCLAF* in human neuroblastoma, we analyzed three neuroblastoma databases (Kocak: GSE45547; SEQC: GSE62564; and NRC: GSE85047). First, survival differences in terms of event-free survival (EFS) and OS were assessed by Kaplan–Meier analysis. Analysis of the three data sets showed that patients with high *PCLAF* mRNA levels had worse OS and EFS (Fig. 1E–H, Supplementary Fig. 1B, C). Next, the relationships between *PCLAF* expression pattern and clinical characteristics were analyzed and showed that the expression of *PCLAF* was positively associated with the clinical stage of neuroblastoma. Intriguingly, from stage I to stage IV, *PCLAF* expression escalated with the clinical stage, but dropped significantly at stage IVs (Fig. 1I-K). This indicated that *PCLAF* is a potential poor prognostic factor in neuroblastoma. In addition, we found that *PCLAF* was significantly overexpressed in children aged over 18 months, in patients at high risk, and in tissue with *MYCN* amplification (Supplementary Fig. 1D-F, Supplementary Tables 4-6). At the same time, multivariate analysis showed that in the SEQC and NRC data sets, the higher the expression of *PCLAF*, the greater the clinical stage. Patients over 18 months of age were associated with more inferior OS and EFS (Supplementary Tables 7, 8). These results indicated that there were differences in the expression of PCLAF in neuroblastoma tissues of diverse pathological types, and high *PCLAF* gene expression in neuroblastoma tissues indicated poor patient prognosis.

### PCLAF can accelerate neuroblastoma proliferation and inhibit cell apoptosis in vitro

**Fig. 2:**
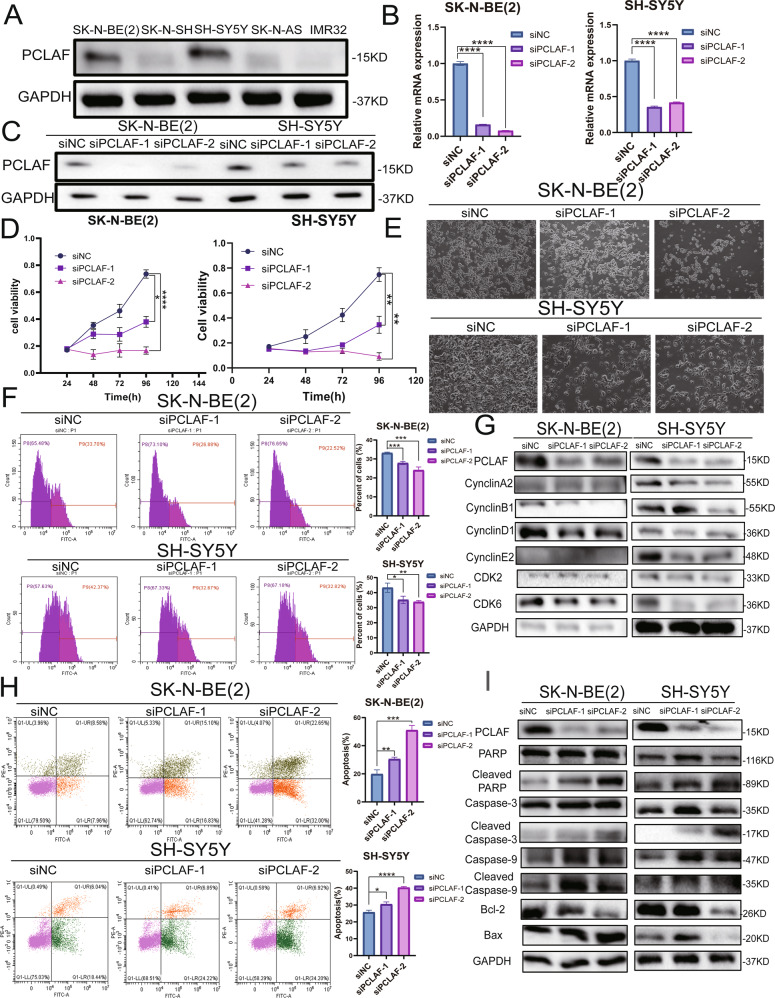
PCLAF is highly expressed and promotes the proliferation of neuroblastoma cells. (**A**) Western blotting examined the protein expression of PCLAF in 5 neuroblastoma cell lines; (**B, C**) Interference efficiency of 2 small interfering RNAs with different sequences on PCLAF in SK-N-BE (2) and SH-SY5Y cell lines, detected by qRT-PCR and Western blotting; (**D**) The in vitro proliferation function of PCLAF was evaluated by CCK-8; (**E**) SK-N-BE(2) and SH-SY5Y cell lines with PCLAF siRNA performed imaging analysis after 48 h; (**F**) The in vitro proliferation function of PCLAF was evaluated by flow cytometry EdU labeling detection; (**G**) Western blotting was used to detect cell proliferation-related proteins; (**H**) PCLAF knockdown led to cell apoptosis in a flow cytometric apoptosis assay; (**I**) Apoptosis related markers were detected by western blot in neuroblastoma cells. (*p < 0.05, **p < 0.01, ***p < 0.001and ****p < 0.0001, and bar graphs represent the mean ± SEM).

To explore whether PCLAF can inhibit the proliferation of neuroblastoma cells, we evaluated PCLAF expression in five neuroblastoma cell lines (SK-N-BE (2), SK-N-AS, SH-SY5Y, SK-N-SH, and IMR32). We selected two cell lines with higher PCLAF expression levels, SH-SY5Y and SK-N-BE (2), for the subsequent experiments (Fig. 2A). First, we tested the silencing efficiency of siRNA targeting *PCLAF* (Fig. 2B, C). We transfected SK-N-BE (2) and SH-SY5Y cell lines with *PCLAF* siRNA, and CCK-8 assay was performed to continuously monitor changes in cell proliferation at 24, 48, 72, and 96 h after transfection. Imaging analysis was performed after 48 h. Interestingly, the number of cells was significantly reduced after silencing PCLAF, indicating that the decreased expression of PCLAF could inhibit the proliferation of neuroblastoma cells (Fig. 2D, E). The results of the EDU experiment showed that after knocking down PCLAF, the percentage of positive signals marked by EDU decreased significantly (Fig. 2F), indicating that the DNA replication activity of the cells had decreased and that the cell proliferation ability was weakened. We further used western blotting and RT-qPCR to detect cell proliferation-related proteins. The decrease in *PCLAF* gene expression significantly downregulated CyclinA2, CyclinB1, CyclinD1, CyclinE2, CDK2, and CDK6 (Fig. 2G, Supplementary Fig. 2A, B), which further showed that downregulation of PCLAF can inhibit the proliferation of neuroblastoma. Apoptosis was evaluated in neuroblastoma cells with PCLAF knockdown after 48 h. The cells were harvested and stained with annexin V-FITC and propidium iodide. Apoptosis analysis showed that the average percentage of apoptotic cells increased significantly after PCLAF knockdown (Fig. 2H), and the results of western blotting detection of apoptosis-related proteins demonstrated that inhibiting the expression of PCLAF can attenuate the expression of antiapoptotic protein BCL-2 and augment the expression of pro-apoptotic proteins PARP, cleaved PARP, caspase 3, cleaved caspase 3, caspase 9, cleaved caspase 9, and BAX (Fig. 2I). In summary, we demonstrated that PCLAF can promote the proliferation of neuroblastoma cells and inhibit cell apoptosis.

### PCLAF knockdown can attenuate the transition of G1/S phase

**Fig. 3:**
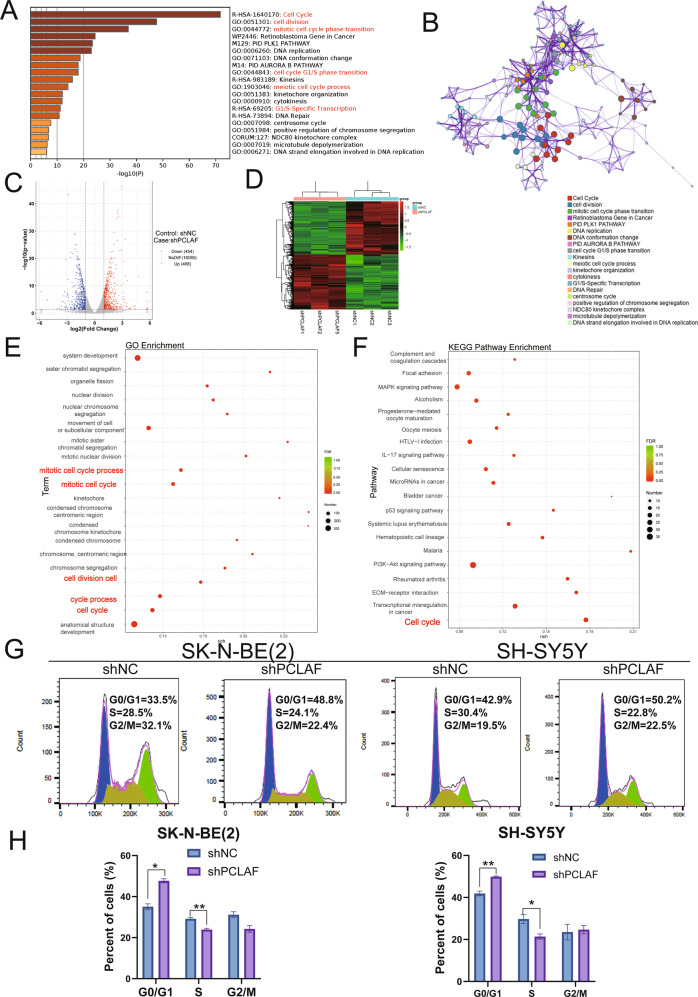
PCLAF triggers pathways related to cell cycle regulation in RNA-seq data. (**A, B**) Heatmap of Gene Ontology (GO) enriched terms and Kyoto Encyclopedia of Genes and Genomes (KEGG) enriched terms colored by *p*-values; (**C**) The volcanic map was drawn according to the gene distribution. The abscissa is log2FoldChange, and the ordinate is -log10(*p* value); (**D**) Cluster heat map of 6 samples. Red represents high expressed genes and green represents low expressed genes; (**E**) The GO enrichment analysis results of differentially expressed genes; (**F**) The KEGG enrichment analysis results of differentially expressed genes. Select the top 20 pathways with the smallest *p* value for display; (**G, H**) Flow cytometry was used to analyze the cell cycle of neuroblastoma cell lines. (*p < 0.05, **p < 0.01, ***p < 0.001and ****p < 0.0001, and bar graphs represent the mean ± SEM).

To explore the relevant molecular mechanisms by which *PCLAF* promoted cell proliferation, we first used the Similar Gene Detection module of GEPIA2 to obtain the top 100 targeted genes related to *PCLAF* based on all TCGA tumor and standard tissue data sets. We performed KEGG (Kyoto Encyclopedia of Genes and Genomes) pathway analysis. We found that these genes were mainly enriched in the “cell cycle,” especially in “cell cycle G1/S phase transition” (Fig. 3A, B), highlighting the hypothesis that *PCLAF* promoted cell proliferation by accelerating the G1/S transition in the cell cycle. To explore the importance of PCLAF in neuroblastoma cell cycle progression, we established SK-N-BE (2) and SH-SY5Y cell lines stably transduced with lentiviruses expressing *PCLAF*-specific short hairpin RNA (shPCLAF) or scrambled shRNA (shNC). After testing the silencing efficiency of shRNA (Supplementary Fig. 3A-C), we performed RNA sequencing (RNA-seq) analysis in SK-N-BE (2) cell lines. Map analysis identified a total of 922 DEGs (|Log2 fold change | > 1 and adjusted *p*-value < 0.05). Among them, 468 genes were upregulated and 454 genes were downregulated (Fig. 3C, D). Gene ontology (GO) analysis results revealed that DEGs were mainly enriched in the cell cycle process, such as “cell division” and “cell cycle.” (Fig. 3E, Supplementary Fig. 3D). Kyoto Encyclopedia of Genes and Genomes pathway analysis showed that cell cycle pathways were also enriched and had the most significant difference (Fig. 3F, Supplementary Fig. 3E). These results indicated that *PCLAF* plays an essential role in neuroblastoma cell cycle progression. At the same time, the cell cycle analysis results showed that reducing the expression of PCLAF in SK-N-BE (2) and SH-SY5Y cell lines could increase the percentage of cells in the G1 phase of the cell cycle (Fig. 3G, H). We further performed a control experiment on a PCLAF nonexpressing cell line (IMR32) and found that compared with SK-N-BE(2) and SH-SY5Y cell lines, knocking down PCLAF had little effect on the G1/S cycle process and the proliferation of IMR32 cells (Supplement Fig. 2C, D). This further indicates that high expression of PCLAF can promote the proliferation of neuroblastoma and G1/S cell cycle transition.

### PCLAF can regulate PTTG1 to promote G1/S cell cycle transition

**Fig. 4:**
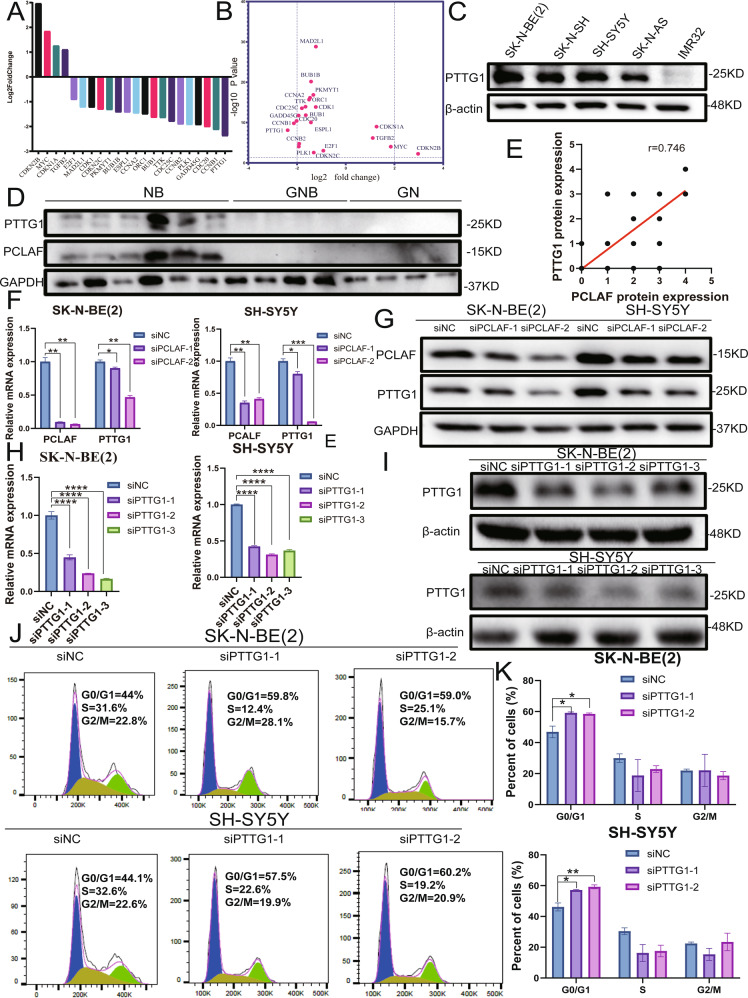
PCLAF can regulate PTTG1 to promote G1/S cell cycle transition. (**A, B**) RNA-seq analyzed the mRNA level of the cell cycle signaling pathway after PCLAF knockdown; (**C**) Western blotting examined the protein expression of PCLAF in 5 neuroblastoma cell lines; (**D**) The expression of PTTG1 and PCLAF in clinical tissues was measured by western blot; (**E**) Spearman correlation analysis of the relationship between PCLAF and PTTG1 in identical clinical tissues (*r* = 0.774, *P* < 0.01); (**F, G**) qRT-PCR and Western blot were used to detect the changes in PTTG1 mRNA and protein levels after interference PCLAF in SK-N-BE(2) and SH-SY5Y cell lines; (**H**, **I**) Interference efficiency of 3 small interfering RNAs with different sequences on PTTG1 in SK-N-BE (2) and SH-SY5Y cell lines, detected by qRT-PCR and Western blotting; (**J, K**) Flow cytometry was used to analyze the cell cycle of neuroblastoma cell lines. (*p < 0.05, **p < 0.01, ***p < 0.001and ****p < 0.0001, and bar graphs represent the mean ± SEM).

To further analyze the cell cycle signaling pathway, we analyzed the results of RNA-seq. We found that the mRNA level of *PTTG1* (pituitary tumor-transforming gene 1) was most significantly decreased after *PCLAF* expression was downregulated (Fig. 4A, B). Therefore, we speculated that PCLAF might regulate the expression of PTTG1. Western blotting revealed that PTTG1 was expressed at higher levels in different neuroblastoma cell lines (Fig. 4C). In addition, we analyzed the expression pattern of PTTG1 in neuroblastoma samples. After analyzing clinical tissue samples by western blotting and TMA, we found that PCLAF and PTTG1 were significantly correlated (*r* = 0.774, *p* < 0.01; Fig. 4D, E, Supplementary Fig. 4A, B). At the same time, we found that there was also a remarkable positive correlation between *PCLAF* and *PTTG1* in three neuroblastoma databases (Supplementary Fig. 4C). In addition, patients with high *PTTG1* mRNA levels had worse OS (*p* = 8.6e − 19) and EFS (*p* = 3.1e − 20) (Supplementary Fig. 4D, E), indicating that *PTTG1* is also a potential prognostic factor in neuroblastoma and may have similar biological functions to *PCLAF*. To prove that PCLAF can regulate the expression of PTTG1, we transfected SH-SY5Y and SK-N-BE (2) cell lines with si*PCLAF*, and PTTG1 expression at both the mRNA and protein level was measured by real-time PCR and western blotting, respectively. This demonstrated that the protein and mRNA levels of PTTG1 were significantly diminished after PCLAF interference (Fig. 4F, G), indicating that PCLAF can promote the expression of PTTG1. It is reported in the literature that reducing the expression of PTTG1 can augment the number of cells in G1 phase and diminish the number of cells in S phase [20]. After we knocked down *PTTG1* in neuroblastoma cells (Fig. 4H, I), we showed that the number of cells in the G1 phase was also significantly increased (Fig. 4J, K). In summary, PCLAF can regulate the expression of PTTG1 to facilitate G1/S cell cycle transition.

### PCLAF-induced PTTG1 activation is mediated by E2F1

**Fig. 5:**
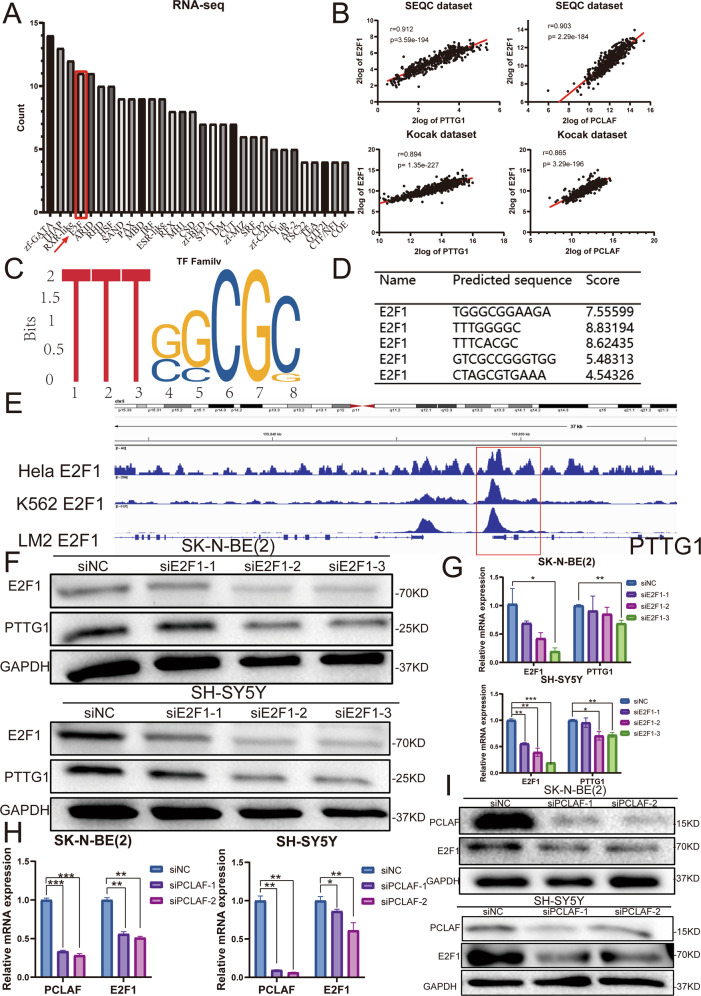
PCLAF regulates PTTG1 by the transcription factor E2F1. (**A**) Family analysis of transcription factors based on the RNA sequencing data; (**B**) Spearman analysis was used to detect the correlations between PCLAF and E2F1 and PTTG1 in the neuroblastoma database; (**C, D**) Prediction of the sequence logo of E2F1 and the binding sites of E2F1 on the PTTG1 promoter region were predicted by JASPAR; (**E**) Graphical representation of enrichment for E2F1 on the regulatory regions of PTTG1 in HeLa, K562, and LM2 cells. Red boxes show regions with E2F1 enrichment; (**F**) The mRNA level of PTTG1 after decreasing E2F1 by siE2F1 in SK-N-BE (2) and SH-SY5Y cell lines; (**G**) The protein levels of PTTG1 after decreasing E2F1 by siE2F1 in SK-N-BE (2) and SH-SY5Y cell lines; (**H**) The mRNA level of E2F1 after decreasing PCLAF by siPCLAF in SK-N-BE (2) and SH-SY5Y cell lines.; (**I**) The protein levels of E2F1 after decreasing PCLAF by siPCLAF in SK-N-BE (2) and SH-SY5Y cell lines. (*p < 0.05, **p < 0.01, ***p < 0.001 and ****p < 0.0001, and bar graphs represent the mean ± SEM).

We demonstrated that PCLAF can upregulate the expression of PTTG1. Transcription factors participate in the transcription initiation complex and play an indispensable role in regulating gene expression. We first analyzed the RNA-seq data to explore whether the DEGs were associated with some transcription events after *PCLAF* is exhausted. Intriguingly, we found that the *E2F* family of transcription factors was one of the most enriched pathways (Fig. 5A). In 2009, Zhou et al. reported that E2F1 could stimulate the expression of PTTG1 in pituitary tumors [21]. Then we analyzed the relationship between *PCLAF* and *PTTG1* genes and *E2F1* in the neuroblastoma database and found that *E2F1* was related to *PCLAF* and *PTTG1* (Fig. 5B). Next, we used the JASPAR database to predict the recognition sequence of E2F1, and further analysis found that E2F1 can bind to multiple sequences in the *PTTG1* promoter region (Fig. 5C, D). Furthermore, public ChIP-sequencing data show striking E2F1 binding peaks in the regulatory area of *PTTG1* in HeLa, K562, and LM2 cell lines (Fig. 5E). In addition, we found that diminishing the expression of E2F1 can reduce the protein and mRNA levels of PTTG1, which indicates E2F1 can alter PTTG1 expression in neuroblastoma by binding to the promoter of *PTTG1* (Fig. 5F, G). The previous results showed that PCLAF was positively correlated with E2F1, and both PCLAF and E2F1 could change the expression of PTTG1. Therefore, we explored whether PCLAF can regulate the expression of PTTG1 through E2F1. After reducing PCLAF expression, we found that E2F1 was dramatically diminished at the mRNA and protein level (Fig. 5H, I). Similarly, we overexpressed E2F1 in SK-N-BE(2) and SH-SY5Y cells (Supplementary Fig. 5A, B). Western blot and q-PCR results showed that the mRNA and protein levels of PTTG1 increased significantly (Supplementary Fig. 5C, D) upon E2F1 overexpression. To further confirm that PCLAF does regulate the expression of PTTG1 through E2F1, we transfected si*PCLAF* and E2F1-flag plasmid into SK-N-BE(2) and SH-SY5Y neuroblastoma cells. We found that overexpression of E2F1 reversed the suppression of PTTG1 levels caused by PCLAF downregulation (Supplementary Fig. 6A). In addition, overexpression of E2F1 and PTTG1 can attenuate the decrease of cell proliferation and the increase of G1/S cells cycle transition that resulted from the silencing PCLAF in SK-N-BE(2) and SH-SY5Y cells (Supplementary Fig. 6B-E). Taken together, these results demonstrated that E2F1 is responsible for PCLAF-mediated activation of PTTG1.

### PCLAF enhances tumor proliferation potential in vivo

**Fig. 6:**
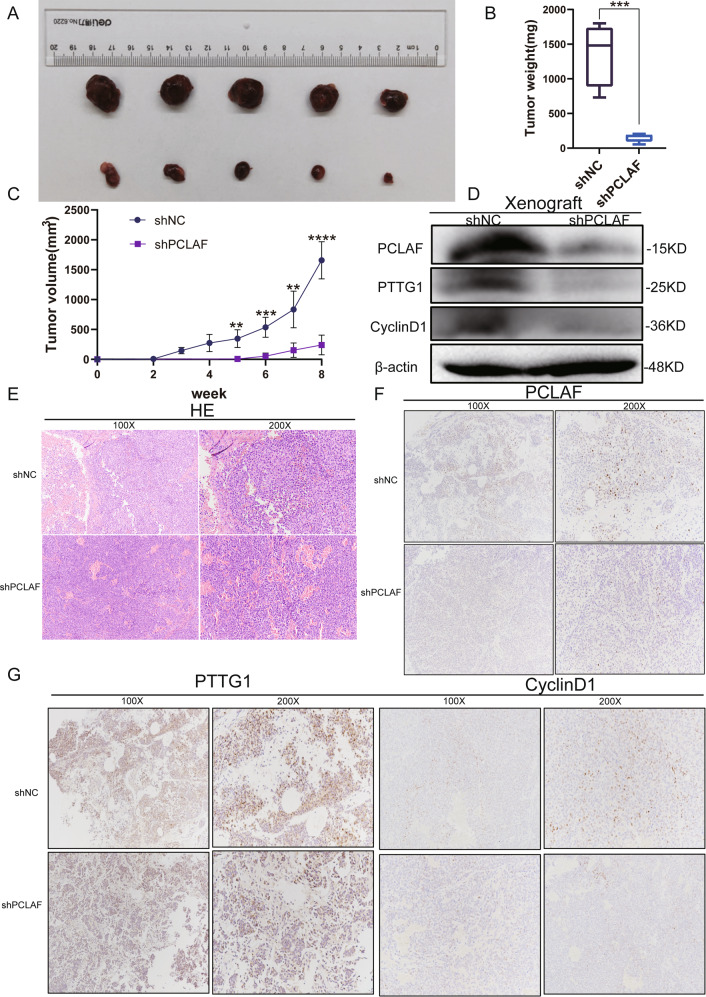
PCLAF knockdown restrained the growth of neuroblastoma in vivo. (**A**) The volume of tumors obtained from xenografts was diminished in the PCLAF-knockdown group by shRNA from the SK-N-BE (2) cell line; (**B, C**) The volume and weight of tumors formed by PCLAF knockdown cells were dramatically smaller than that of the control; (**D**) The expression of PCLAF, PTTG1 and CyclinD1 were examined by western blot in tumors obtained from xenografts; (**E**) H&E staining of subcutaneous tumors in BALB/c nude mice; (**F, G**) Immunohistochemical staining of subcutaneous tumors on target of PCLAF, PTTG1 andCyclinD1 separately. (*p < 0.05, **p < 0.01, ***p < 0.001 and ****p < 0.0001, and bar graphs represent the mean ± SEM).

Our in vitro experiments demonstrated that PCLAF can promote the proliferation of neuroblastoma cells. Therefore, to further study the role of PCLAF in vivo, we established a subcutaneous xenograft nude mouse model. After 8 weeks, tumors derived from shNC cells at the subcutaneous implantation site were heavier and grew faster than tumors derived from sh*PCLAF* cells (Fig. 6A-C). In xenograft mice, inhibition of PCLAF expression could also reduce PTTG1 and cyclin D1 (Fig. 6D). H&E staining showed that the tumors of shNC mice had more active mitosis than those of sh*PCLAF* mice (Fig. 6E). Moreover, western blotting and IHC also revealed the effect of PCLAF knockdown on PTTG1 and cyclin D1 (Fig. 6 F, G). Taking these results together, our study demonstrated that PCLAF could promote the proliferation and growth of neuroblastoma in vivo.

## Discussion

PCLAF has been identified as a PCNA binding partner and is involved in many human malignancies [12, 22]. In vitro studies have shown that PCLAF participates in DNA repair through interactions with PCNA. In vivo studies have also confirmed the role of PCLAF in cancer and stem cells independent of PCNA function [23, 24]. This study confirmed the expression, clinical significance, and functional role of PCLAF in neuroblastoma for the first time.

Our study found that the expression of PCLAF in different pathological types of neuroblastoma has apparent differences, and the presentation of PCLAF in neuroblastoma is higher than that in GNB and GN. At the same time, *PCLAF* expression was correlated with age at diagnosis, histological type, Shimada pathological type, risk grade, and clinical stage, and OS and EFS with high *PCLAF* expression were worse in neuroblastoma databases. These data demonstrate that PCLAF may be a factor leading to differences in the prognosis of patients with neuroblastoma.

PCLAF has a considerable role in cell cycle regulation and DNA replication. Studies revealed an increase in the number of cells in G1 phase and a decrease in the number of cells in S phase following depletion of PCLAF. PCLAF promotes the proliferation of cancer cells in a variety of human malignancies [16, 25–27]. A recent paper has highlighted the key role of PAF (PCLAF) in modulating gene expression patterns and G1/S progression controlled by the DREAM complex in lung cancer [27]. We analyzed the results of RNA-seq and found that knocking down *PCLAF* in neuroblastoma can reduce the mRNA expression levels of some genes in the DREAM complex, such as FOXM1, CCNA2, BUB1, CCNB2, PLK1, CCNB1 and B-MYB, indicating the DREAM complex also played a part in the PCLAF regulation of neuroblastoma G1/S process. Since the DREAM complex (B-MYB, FOXM1) have previously been shown to be essential for neuroblastoma cell proliferation and survival [28, 29], this further suggests that PCLAF plays an important role in neuroblastoma cell proliferation and survival. Herein, we found that reducing the expression of PCLAF could restrict the proliferation of neuroblastoma cells, leading to cell arrest in G1 phase and promoting neuroblastoma apoptosis.

Although previous studies have reported that PCLAF has an important role in the cell cycle, the mechanism by which PCALF regulates cell cycle progression remains unclear. Most importantly, the mechanism by which PCLAF promotes neuroblastoma proliferation is also unclear. To identify the downstream targets of PCLAF, we performed RNA-seq analysis on neuroblastoma cells stably transfected with *PCLAF* shRNA. Through the results of RNA-seq data, we found that the mRNA level of *PTTG1* in the cell cycle pathway was decreased most significantly. Hence, we suspect that PCLAF may affect the cell cycle process by regulating *PTTG1*. To our knowledge, this is the first report demonstrating PCLAF can regulate the expression of PTTG1 in neuroblastoma cells.

*PTTG1* is overexpressed in most human tumors and plays a role in cell replication, DNA damage/repair, organ development, and metabolism, and can accelerate tumor cell proliferation, migration, invasion, and angiogenesis [30–33]. Our research found that *PTTG1* is expressed at higher levels in neuroblastoma. After analyzing clinical tissue samples and public databases, we found that PCLAF and PTTG1 have a strikingly positive correlation, and patients with high *PCLAF* mRNA levels have worse OS and EFS. Subsequent experiments confirmed our hypothesis that reducing the expression of *PTTG1* can also lead to an increase in the number of cells in G1/G0. In our study, we demonstrated that PCLAF can regulate *PTTG1* to promote G1/S cell cycle transition.

Transcription factors have a vital role in the regulation of gene expression. For example, we found that the E2F family of transcription factors is one of the most enriched pathways in RNA sequencing data. At the same time, there are reports that E2F1 can enhance the expression of PTTG1 in pituitary tumors, glioma, and adrenocortical carcinoma [21, 34, 35]. In neuroblastoma databases, we also found that *E2F1* is positively correlated with *PCLAF* and *PTTG1*. We confirmed that PCLAF could regulate the expression of *PTTG1* in neuroblastoma by affecting the E2F1 transcription factor. However, the detailed molecular mechanism of PCLAF-induced E2F1 expression remains unclear, and further mechanistic studies are required.

In summary, our research shows that PCLAF is strongly associated with various adverse clinicopathological parameters and is a potential prognostic indicator in neuroblastoma. In addition, PCLAF can regulate the expression levels of *PTTG1* through E2F1 and promote the proliferation of neuroblastoma cells. Our results provide a new perspective for the study of neuroblastoma and demonstrate a theoretical basis for the development of new anti-tumor therapeutic drugs targeting PCLAF. However, additional mechanisms need to be explored, such as the specific mechanism by which PCLAF affects E2F1 expression and how PTTG1 affects neuroblastoma cell cycle progression.

## Supplementary information

Supplementary Material

Supplementary Figure 1

Supplementary Figure 2

Supplementary Figure 3

Supplementary Figure 4

Supplementary Figure 5

Supplementary Figure 6

Original Data File

aj-checklist

## Data Availability

All data included in this study are available by contacting the corresponding authors.
