# Supplementary material for: PCLAF promotes neuroblastoma G1/S cell cycle progression via the E2F1/PTTG1 axis
Source: Cell Death Dis. 2022 Feb 24;13(2):178. doi: 10.1038/s41419-022-04635-w (PMC8873510; doi:10.1038/s41419-022-04635-w)
Supplement: Supplementary file 1 — Supplementary Material [file 41419_2022_4635_MOESM1_ESM.docx]

**Supplementary Figure 1**

**The prognostic value of PCLAF and relationship between PCLAF and clinical information in the Kocak, SEQC and NRC neuroblastoma databases**

(A) Preliminary observation of the expression of PCLAF in 70 cases of neuroblastoma TMA; (B, C) Kaplan-Meier analysis of OS and EFS for the Kocak data set(n= 476, 173 patients without survival information was not included in the dataset); (D) In dataset Kocak, age at diagnosis more than 18 months have higher PCLAF expression and differential expression of PCLAF between MYCN amplified and non-amplified; (E) Box plot of PCLAF expression between high-risk and low-risk groups, differential expression of PCLAF between MYCN amplified and non-amplified in dataset SEQC; (F) In dataset NRC, box plots of differential expression of PCLAF between MYCN amplified and non-amplified.

**Supplementary Figure 2**

(A, B) qRT-PCR analysis of proliferation related proteins in PCLAF knockdown cells.

(C) Flow cytometry was used to analyze the cell cycle of IMR32 cell line.

(D) Flow cytometry EdU labeling detection was used to evaluate the proliferation function of IMR32 cell line after knocking down the PCLAF. (*p < 0.05, **p < 0.01, ***p < 0.001and ****p < 0.0001, and bar graphs represent the mean ± SEM)

**Supplementary Figure 3**

(A-C) The knockdown efficiency of the lentivirus tool on PCLAF in SK-N-BE (2) and SH-SY5Y cell lines; (D) The GO enrichment analysis results of differentially expressed genes; (E) The KEGG enrichment analysis results of differentially expressed genes. (*p < 0.05, **p < 0.01, ***p < 0.001and ****p < 0.0001, and bar graphs represent the mean ± SEM)

**Supplementary Figure 4**

(A, B) Spearman analysis was used to detect the correlations between PCLAF and PTTG1 in 70 cases of neuroblastoma TMA; (C)Spearman analysis was used to detect the correlations between PCLAF and PTTG1 in the neuroblastoma database(p=0.746); (D, E) Kaplan-Meier analysis of OS and EFS for the SEQC data set and the Kocak data set, based on PCLAF expression with the log-rank test p value indicated. (*p < 0.05, **p < 0.01, ***p < 0.001 and ****p < 0.0001, and bar graphs represent the mean ± SEM)

**Supplementary Figure 5**

(A, B) qRT-PCR and Western blot were used to detect the mRNA and protein level of E2F1 after overexpression of E2F1 in in SK-N-BE (2) and SH-SY5Y cell lines. (C, D) qRT-PCR and Western blot were performed to detect the mRNA and protein level of E2F1 and PTTG1 after overexpression of E2F1 in in SK-N-BE (2) and SH-SY5Y cell lines. (*p < 0.05, **p < 0.01, ***p < 0.001 and ****p < 0.0001, and bar graphs represent the mean ± SEM)

**Supplementary Figure 6**

(A) Cells overexpressing E2F1-Flag were transfected with NC or siPCLAF-2 for 48 h. Cell lysates were analyzed by western blot using the indicated antibodies. (B, C) Proliferation assay was performed by flow cytometry EdU labeling detection, the proliferation rates in both PCLAF silencing cell lines were decreased and overexpress E2F1 at this basis could rescue the proliferation status. (D, E) Cell cycle assay was performed by flow cytometry, overexpression of E2F1 can attenuate the increase of G1/S cells cycle transition that resulted from the silencing PCLAF in SK-N-BE(2) and SH-SY5Y cells. (*p < 0.05, **p < 0.01, ***p < 0.001 and ****p < 0.0001, and bar graphs represent the mean ± SEM)

**Supplemental Table 1**

**siRNA sequences**

| **siRNA** | **Sequence** |
| --- | --- |
| **siPCLAF-1** | **CGACATCAGTTTCATCGAG** |
| **siPCLAF-2** | **CATGGTGCGGACTAAAGCA** |
| **siPTTG1-1** | **GAAGACTGTTAAAGCAAAA** |
| **siPTTG1-2** | **GATGATGCCTATCCAGAAA** |
| **siPTTG1-3** | **GATGGGAGATCTCAAGTTT** |
| **siE2F1-1** | **CTGCAGATATATCTCAAGA** |
| **siE2F1-2** | **GGGAGAAGACTCGGTATGA** |
| **siE2F1-3** | **GCGATCTCTTCGACTCCTA** |
| **shPCLAF** | **CATGGTGCGGACTAAAGCA** |

**Supplemental Table 2**

**Primers designed for PCR**

| **Genes** | **Primers** |
| --- | --- |
| **PCLAF** | **Forward GGCAAGGAGGACAAATACGCA** |
|  | **Reverse TGTGCCCACCATGATTCTATCC** |
| **PTTG1** | **Forward GCTTTGGGAACTGTCAACAGAGC** |
|  | **Reverse CTGGATAGGCATCATCTGAGGC** |
| **E2F1** | **Forward GGACCTGGAAACTGACCATCAG** |
|  | **Reverse CAGTGAGGTCTCATAGCGTGAC** |
| **CyclinA2** | **Forward CTCTACACAGTCACGGGACAAAG** |
|  | **Reverse CTGTGGTGCTTTGAGGTAGGTC** |
| **CyclinB1** | **Forward GACCTGTGTCAGGCTTTCTCTG** |
|  | **Reverse GGTATTTTGGTCTGACTGCTTGC** |
| **CyclinD1** | **Forward TCTACACCGACAACTCCATCCG** |
|  | **Reverse TCTGGCATTTTGGAGAGGAAGTG** |
| **CyclinE2** | **Forward CTTACGTCACTGATGGTGCTTGC** |
|  | **Reverse CTTGGAGAAAGAGATTTAGCCAGG** |
| **CDK2** | **Forward ATGGATGCCTCTGCTCTCACTG** |
|  | **Reverse CCCGATGAGAATGGCAGAAAGC** |
| **CDK6** | **Forward GGATAAAGTTCCAGAGCCTGGAG** |
|  | **Reverse GCGATGCACTACTCGGTGTGAA** |
| **GAPDH** | **Forward GTCTCCTCTGACTTCAACAGCG** |
|  | **Reverse ACCACCCTGTTGCTGTAGCCAA** |

**Supplemental Table 3**

**Chip sequencing data**

| **Experiment** | **Datasets GEO** | **Datasets ENCODE** | **Initial Data** |
| --- | --- | --- | --- |
| **Hela** | | | |
| **E2F1** | **GSM935484** |  | **SRR502355.sra SRR502356.sra** |
| **Input** | **GSM509051** |  | **SRR036650.sra**  **SRR036651.sra SRR036652.sra SRR036653.sra SRR036654.sra** |
| **K562** | | | |
| **E2F1** |  | **Replicat 1: ENCLB809EWT** | **ENCFF749JUG.fastq ENCFF942QFN.fastq** |
| **Input** | **GSM777645** |  | **SRR332096.sra** |
| **LM2** | | | |
| **E2F1** | **GSM2501567** |  | **SRR5282135.sra** |
| **Input** | **GSM2501570** |  | **SRR5282138.sra** |

**Supplementary Table 4**

**Correlation analysis between clinical characteristics and expression of PCLAF in the SEQC data set.**

| Covariates | Total | PCLAF | | X^2^ | P |
| --- | --- | --- | --- | --- | --- |
|  |  | High | Low |  |  |
| Age at diagnosis  ≥18 months  <18 months  Clinical stages  I–II IV–S  II–IV  Risk  Low risk  High risk  MYCN state  Amplification  Non-amplification | 198  300  252  246  322  176  92  401 | 66  53  30  89  37  82  52  66 | 132  247  222  157  285  94  40  335 | 16.098  40.333  77.096  65.971 | <0.001  <0.001  <0.001  <0.001 |

**Supplementary Table 5**

**Correlation analysis between clinical characteristics and expression of PCLAF in the NRC data set.**

| Covariates | Total | PCLAF | | X^2^ | P |
| --- | --- | --- | --- | --- | --- |
|  |  | High | Low |  |  |
| Age at diagnosis  ≥18 months  <18 months  Clinical stages  I–II IV–S  II–IV  MYCN state  Amplification  Non-amplification | 134  144  113  167  55  222 | 31  12  6  37  21  21 | 103  132  107  130  34  201 | 11.629  14.714  28.270 | <0.001  <0.001  <0.001 |

**Supplementary Table 6**

**Correlation analysis between clinical characteristics and expression of PCLAF in the Kocak data set.**

| Covariates | Total | PCLAF | | X^2^ | P |
| --- | --- | --- | --- | --- | --- |
|  |  | High | Low |  |  |
| Age at diagnosis  ≥18 months  <18 months  Clinical stages  I–II IV–S  II–IV  MYCN state  Amplification  Non-amplification | 134  144  335  235  70  495 | 79  110  74  115  50  136 | 77  334  261  120  20  359 | 35.795  44.914  53.653 | <0.001  <0.001  <0.001 |

**Supplementary Table 7**

**Multivariable analyses in the SEQC data set**

| **Covariates** | **OS** |  | **EFS** |  |
| --- | --- | --- | --- | --- |
|  | **HR (95%CI)** | **P** | **HR (95%CI)** | **P** |
| **PCLAF expression**  (high vs. low) | 2.889（1.946-4.287） | <0.0001 | 3,041(2.048-4.516) | <0.0001 |
| **Clinical stages**  (I–II IV–S vs. III–IV) | 6.230（2.993-12.971） | <0.0001 | 6.443(3.085-13.457) | <0.0001 |
| **Age at diagnosis**  (< >18 months) | 3．588（2.136-6.025） | <0.0001 | 3.198(1.902-5.379) | <0.0001 |

**Supplementary Table 8**

**Multivariable analyses in the NRC data set**

| **Covariates** | **OS** | |  | **EFS** |  |
| --- | --- | --- | --- | --- | --- |
|  | **HR (95%CI)** | **P** | | **HR (95%CI)** | **P** |
| **PCLAF expression**  (high vs. low) | 2.233（1.360-3.665） | 0.001 | | 2.357(1.553-3.632) | <0.0001 |
| **Clinical stages**  (I–II IV–S vs. III–IV) | 11.861（3.509-40.094） | <0.0001 | | 4.078(2.012-8.269) | <0.0001 |
| **Age at diagnosis**  (< >18 months) | 2.453（1.309-4.596） | 0.005 | | 1.763(1.054-2.949) | 0.031 |

**Supplementary Materials and Methods**

**Tissue Specimens**

In addition, all the patients selected for this study had their tumor samples removed by surgery. The fresh tissue samples were immediately snap frozen in liquid nitrogen and stored at −80°C for tissue microarray (TMA) and western blotting experiments. The ethics committee of our institution approved the study and the consent procedure of Xinhua Hospital affiliated to Shanghai Jiaotong University School of Medicine.

**Tissue microarray and immunohistochemistry (IHC)**

Immunohistochemical staining was performed with specific antibodies against PCLAF (1:150; CST, USA) and PTTG1 (ab79546, 1:100, Abcam). We selected three random fields to observe the intensity of immunoreactivity in tissue sections by microscopy, which were evaluated by two pathologists blinded to the clinicopathological characteristics of the tumors. Immunohistochemistry results were measured on a specialized scale from 0 to 4: 0 (no detectable stained tumor), negative expression; 1 (less than 10% positive cells), weakly positive expression; 2 (11%–30% of stained cells), intermediately positive expression; 3 (31%–50% of cells stained positive), moderately positive expression; and 4 (51%–100% of cells stained positive), strongly positive expression.

**Cell culture and transfection**

Neuroblastoma cell lines were cultured in F12 medium (Gibco, USA) and Eagle’s minimal essential medium (Gibco, USA) supplemented with 10% heat-inactivated fetal bovine serum (Gemini, USA). All cell lines were incubated at 37°C in a humid incubator with air containing 5% CO_2_. The cells were transfected with siRNA (siPCLAF, siPTTG1, siE2F1, and negative control siNC; Table S1) using Lipofectamine™ RNAimax (Thermo Fisher Scientific, USA) according to the manufacturer's instructions. siRNAs were synthesized by Riobio Biotechnology (Guangzhou, China).

**Western blotting**

Primary antibodies against PCLAF (81533S; 1:1000), PTTG1 (13445S; 1:1000), E2F1 (3742S; 1:1000), GAPDH (2118s; 1:2000), β-actin (3700S; 1:1000), cyclin D1 (2978s; 1:1000), cyclin A2 (4656S; 1:1000), cyclin B1 (12231S; 1:1000), cyclin E2 (4132S; 1:1000), CDK2 (18048S; 1:1000), CDK6 (13331S; 1:1000), BAX (14796S; 1:1000), and BCL-2 (15071S; 1:1000) were purchased from Cell Signaling Technology (Beverly, USA). Apoptosis Antibody Sampler Kit (9915T; 1:1000) was also purchased from Cell Signaling Technology. The quantitative results were examined by Image J software (X64, v. 2.1.4).

**Quantitative real-time PCR**

TRIzol reagent (Invitrogen, USA) was used to extract total RNA from cells. Reverse transcription reactions were performed with a reverse transcription kit (Cat. RR036A; TAKARA, Japan). SYBR Green Master Mix (11198ES03; Yeasen, Shanghai, China) was used for quantitative real-time PCR (qRT-PCR). The primer sequences are detailed in Table S2. Relative mRNA expressions levels were determined with the internal control *GAPDH* using the ^2−ΔΔ^CT method.

**PCLAF-related gene enrichment analysis**

Metascape (http://metascape.org) is a well-maintained gene list analysis tool for gene annotation and analysis. In this study, Metascape was used to execute pathway and handle enrichment analysis of PCLAF-related genes. It is an automatic meta-analysis tool that can understand a set of orthogonal target discovery common and unique paths in research.

**RNA sequencing analysis**

Library preparation for clustering and sequencing, transcriptome sequencing, and data analysis were completed by Personalbio Bioinformatics Co., Ltd. (Nanjing, China). Three biological replicates were generated for each sample. First, we used HTSeq (0.9.1) statistical data to align the read count value of each gene, which was used as the primary expression level of the gene, and then FPKM (fragments per kilo bases per million fragments) was performed for normalizing the expression levels. The prerequisites for screening differentially expressed genes (DEGs) were as follows: expression difference multiple | log2FoldChange |> 1 and a significant p-value of <0.05. We used DESeq (1.30.0) to analyze DEGs.

**Chip sequencing analysis process**

Raw reads were screened to acquire high-quality clean reads by eliminating sequencing adapters, short reads (length <35 bp), and low-quality reads, which were first preprocessed by Cutadapt (v1.9.1) and Trimmomatic (v0.35). FastQC was used to ensure high-quality data. The clean reads were aligned to the mouse genome (assembly GRCm38) using the Bowtie2 (v2.2.6) software. The MACS (Model-based Analysis of ChIP-Seq) algorithm was used to determine enriched peak regions by applying a p-value cutoff of 0.01. Integrative Genomics Viewer (IGV) software was used to present the visualization of ChIP peaks (version 2.3.91).

**Animal experiments**

The nude mice were divided into two groups (five mice per group). Then 200 μl Matrigel (Corning, New York, USA) containing 1 × 10^7^ shPCLAF or shNC SK-N-BE (2) cells were subcutaneously injected into the left armpits of nude mice. The tumor volume was monitored with a caliper every 7 days by measuring the length and width of the tumor. Tumor volume was assessed using the following formula: volume (mm^3^) = (length × width ^2^)/2. The mice were then killed and the tumors was removed and fixed in buffered formaldehyde for immunohistochemical staining.

**Cell viability analysis**

Cells were seeded in 96-well plates at a density of 5 × 10^3^ cells and cultured for 4 days. After the cells had adhered to the wells, CCK-8 assay was performed for the first time and then every 24 h according to the manufacturer's instructions. Absorbance at 450 nm and 630 nm were determined with a microplate reader.
