# Supplementary material for: PCLAF promotes neuroblastoma G1/S cell cycle progression via the E2F1/PTTG1 axis
Source: Cell Death Dis. 2022 Feb 24;13(2):178. doi: 10.1038/s41419-022-04635-w (PMC8873510; doi:10.1038/s41419-022-04635-w)

Original western blots

Figure-1 B

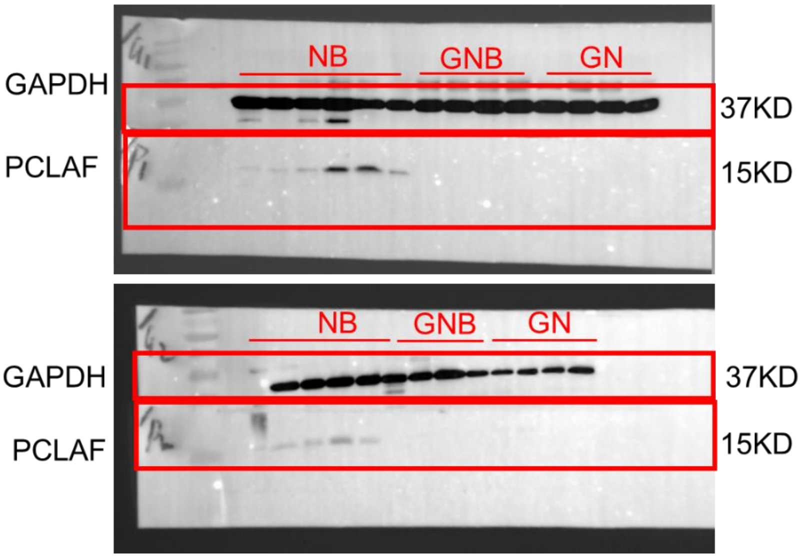

Figure-2 A

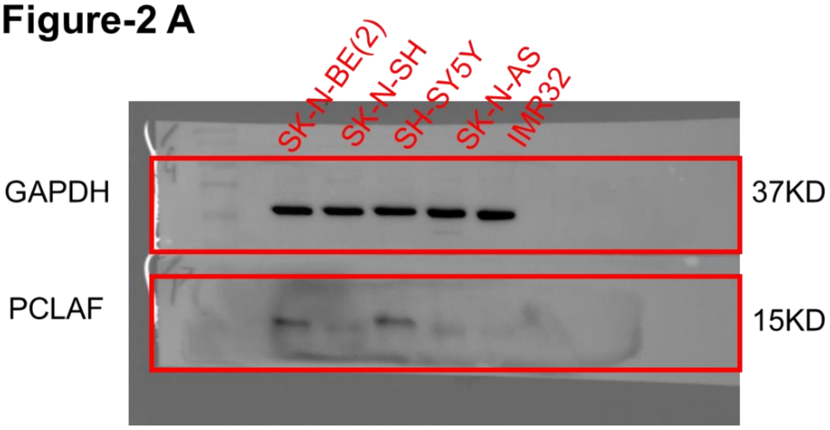

Figure-2 C

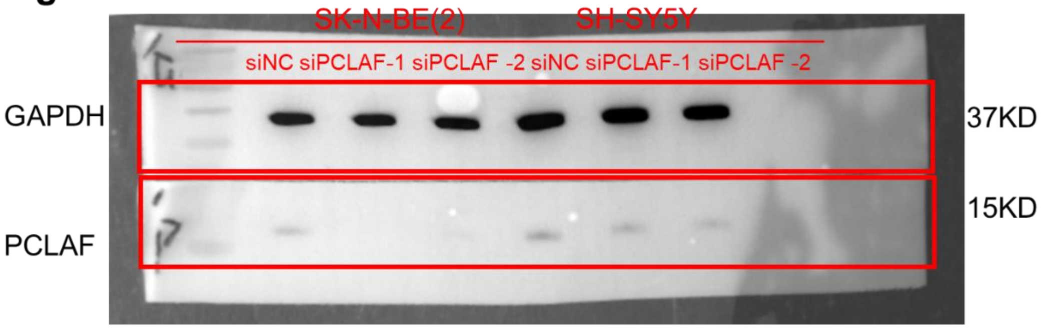

Figure-2 G SK-N-BE(2)

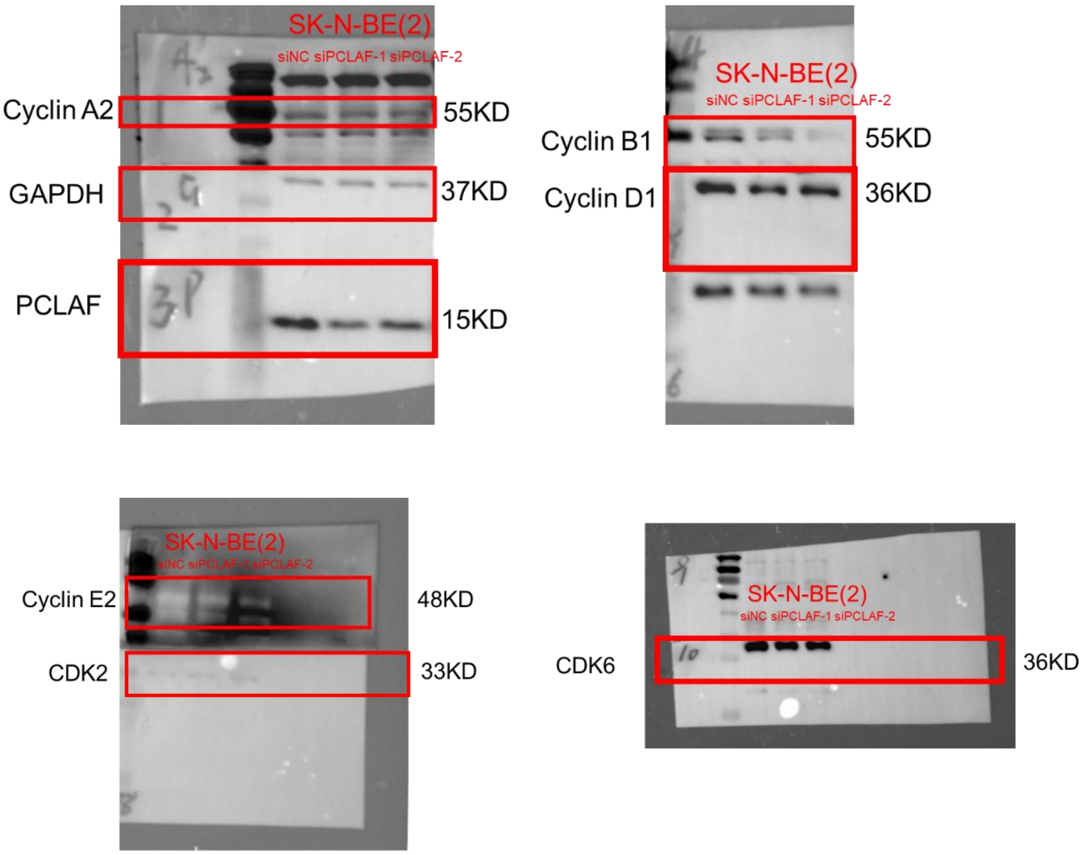

Figure-2 G SH-SY5Y

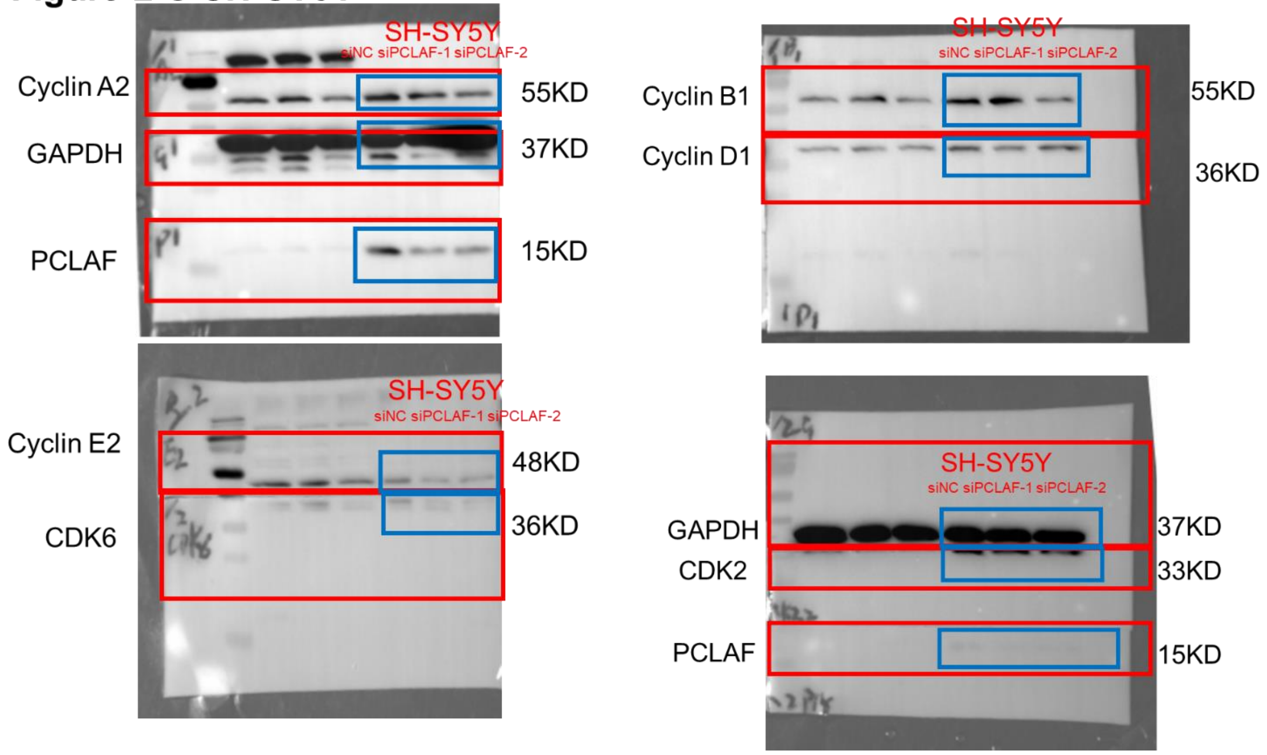

Figure-2 I SK-N-BE(2)

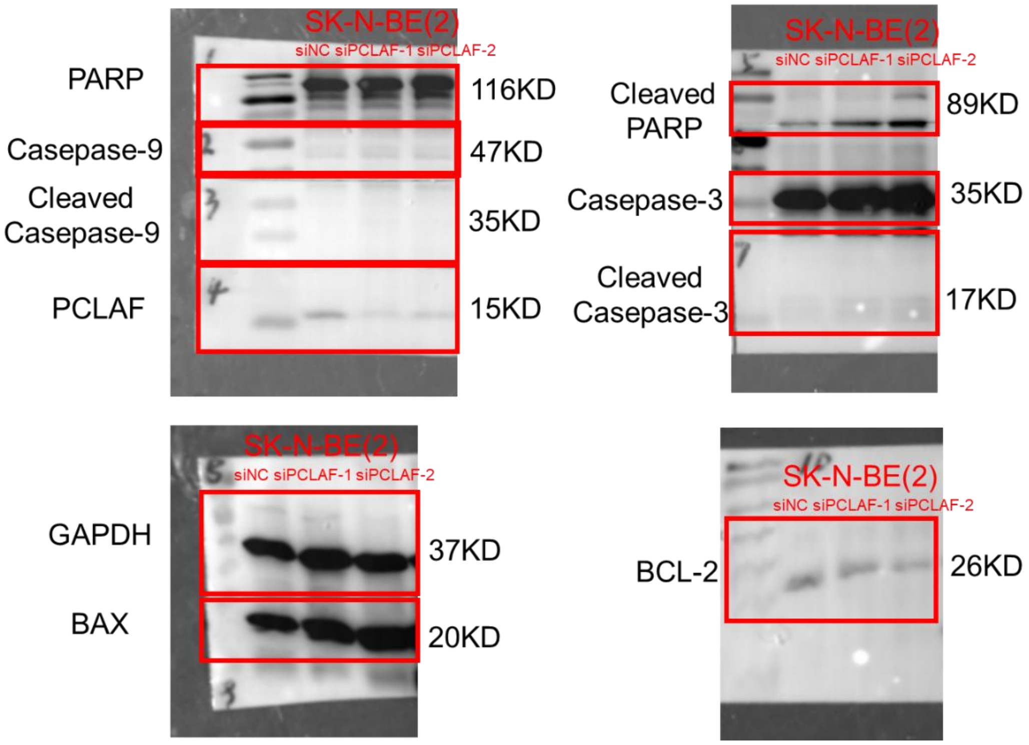

Figure-2 I SH-SY5Y

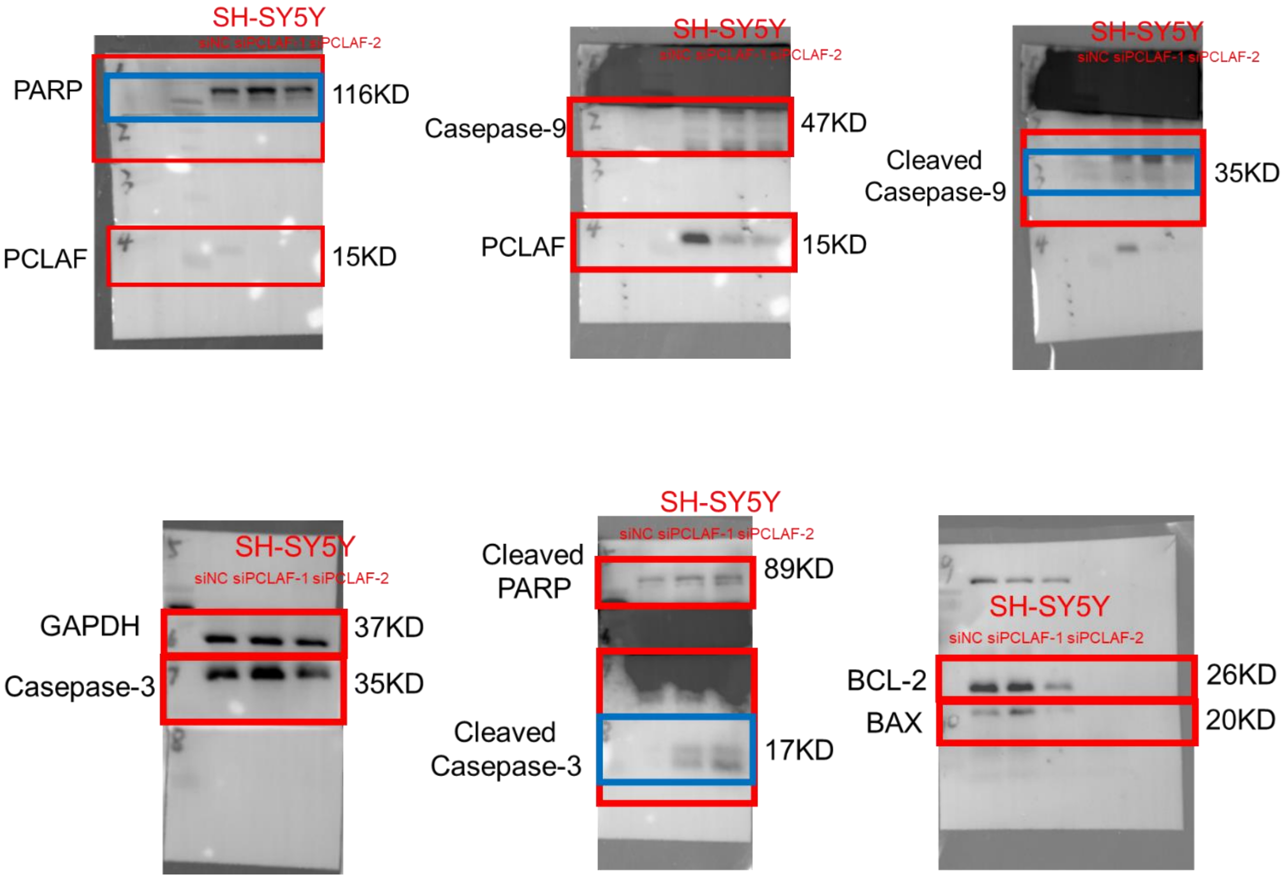

Figure-4 C

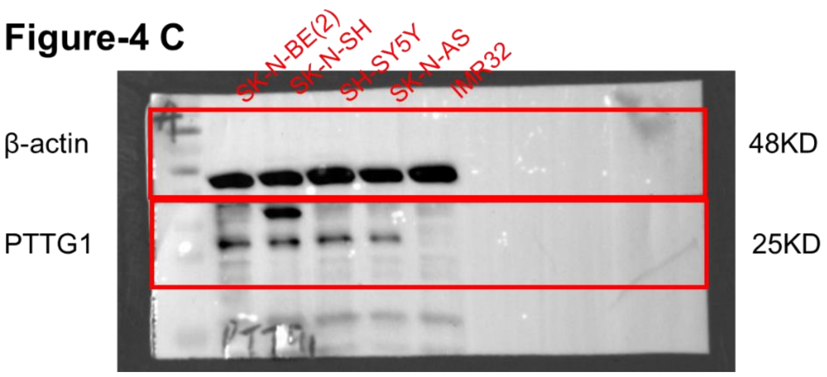

Figure-4 D

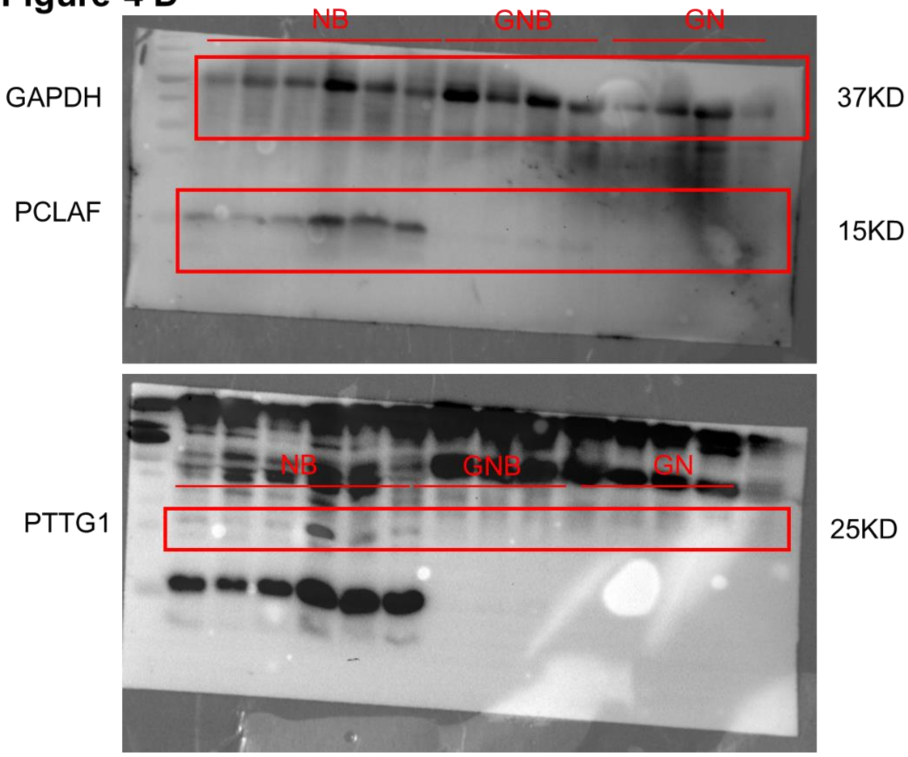

Figure-4 G

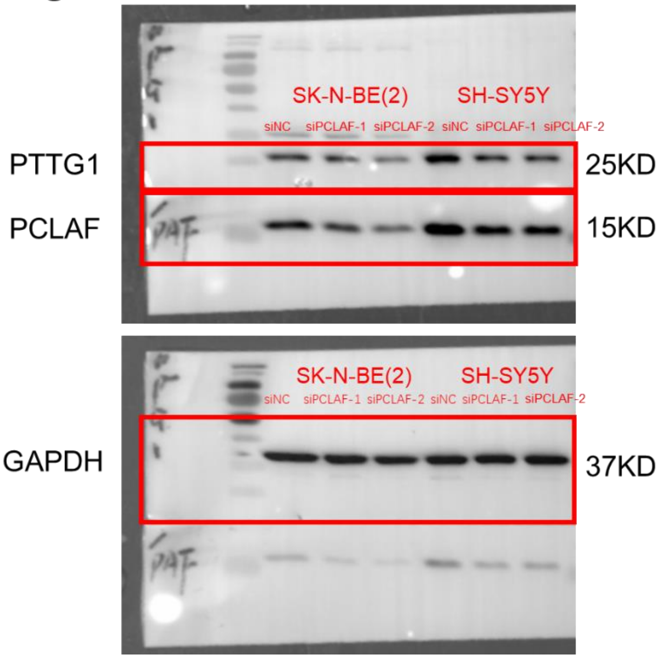

Figure-4 I SK-N-BE(2)

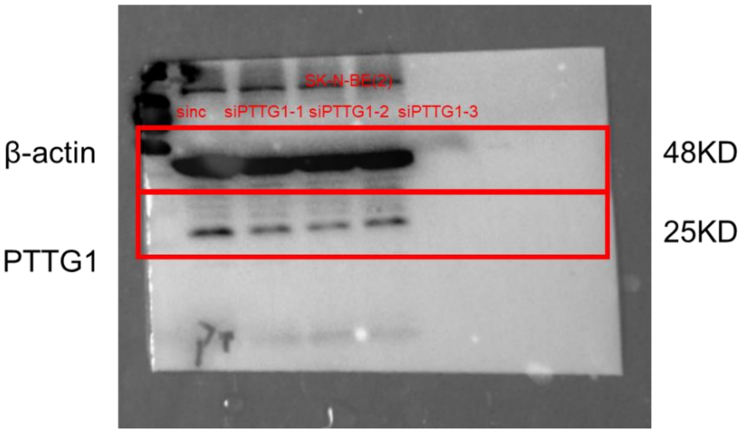

Figure-4 I SH-SY5Y

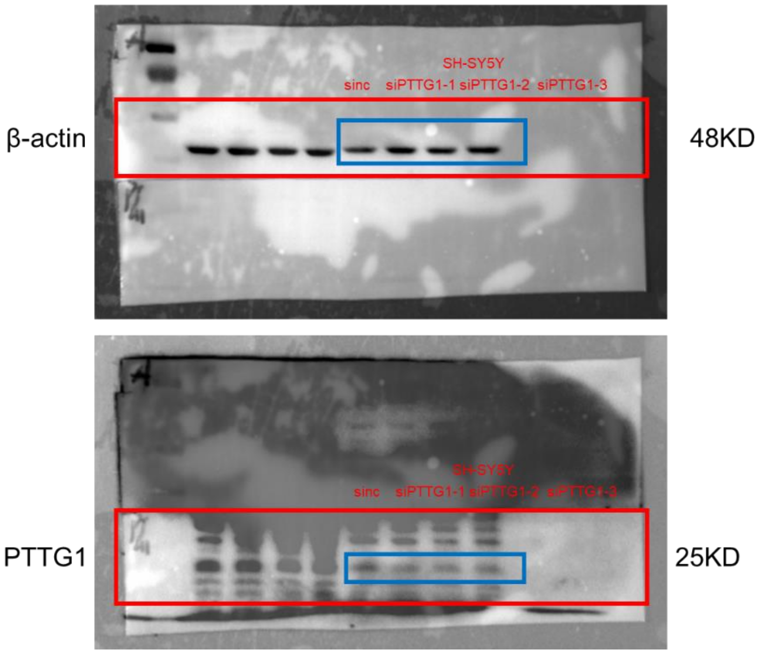

Figure-5 F

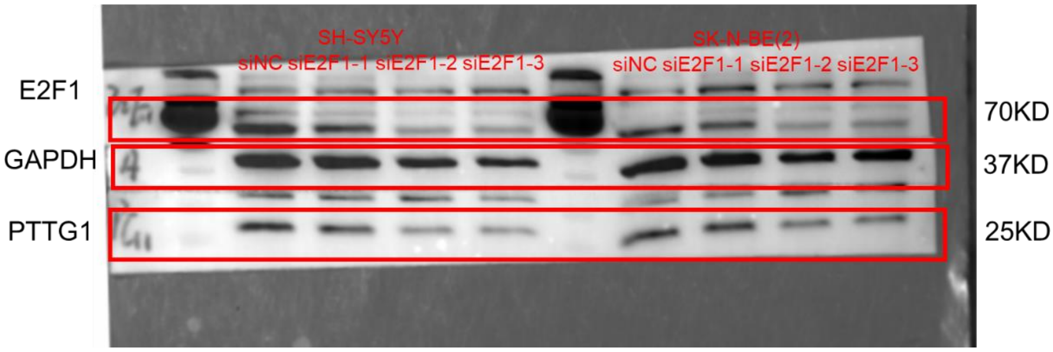

Figure-5 I SK-N-BE2

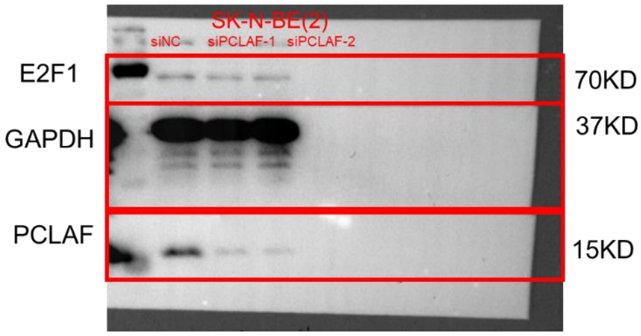

Figure-5 I SH-SY5Y

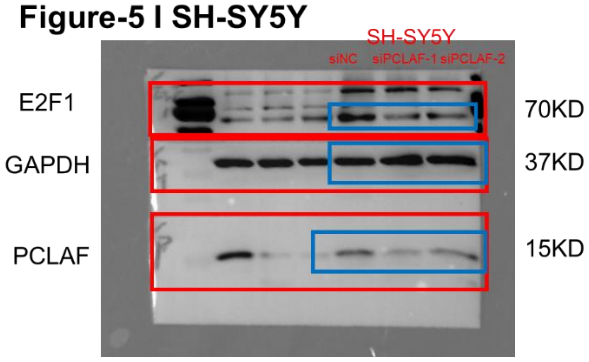

Figure-6 D

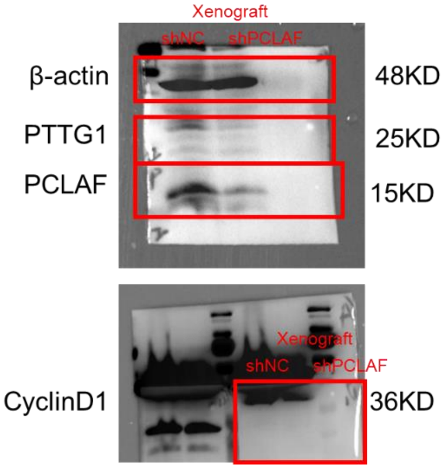

Supplementary Figure-3 C

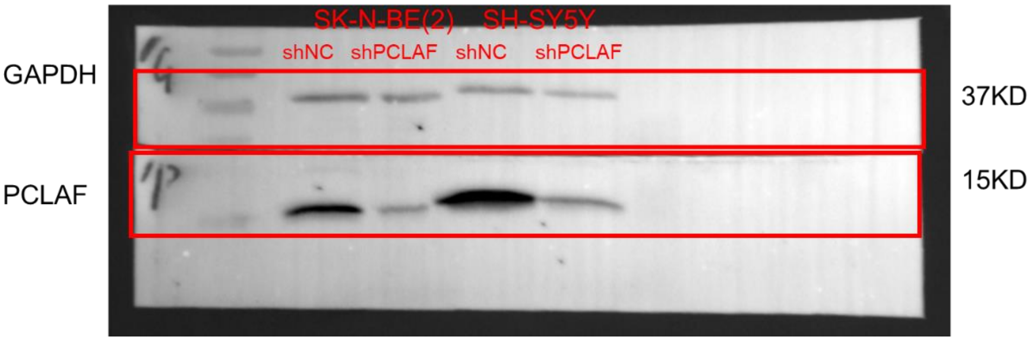

Supplementary Figure-5 B

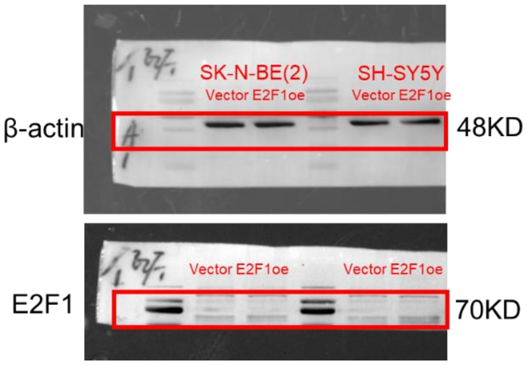

Supplementary Figure-5 D

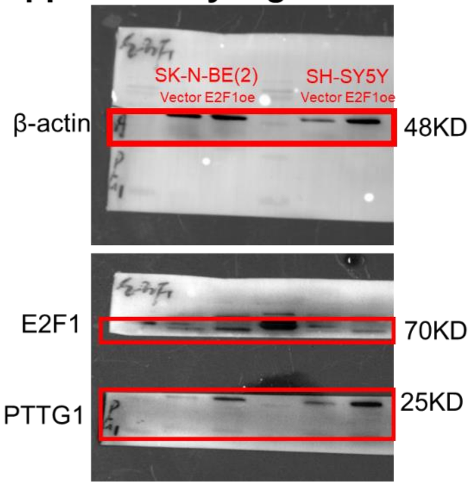

Supplementary Figure-6 A

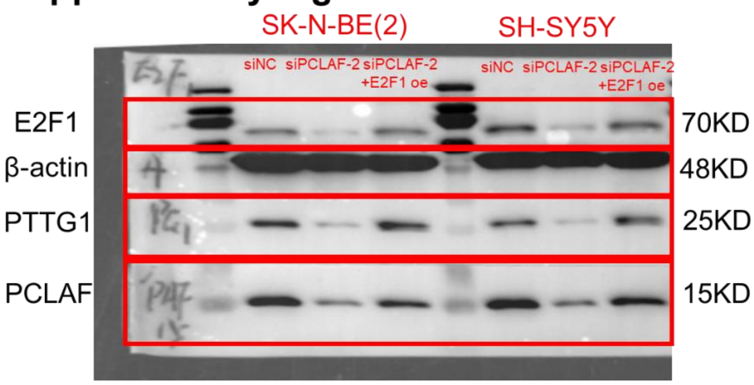

Supplement: Supplementary file 8 — Original Data File [file 41419_2022_4635_MOESM8_ESM.pdf]
